# Supplementary material for: Yield of genetic association signals from genomes, exomes and imputation in the UK Biobank
Source: Nat Genet. 2024 Sep 25;56(11):2345–51. doi: 10.1038/s41588-024-01930-4 (PMC11549045; doi:10.1038/s41588-024-01930-4)
Supplement: Supplementary file 1 — Supplementary Note, Supplementary Figs. 1–13 and Supplementary Tables 1–8. [file 41588_2024_1930_MOESM1_ESM.pdf]

nature genetics

Supplementary information https://doi.org/10.1038/s41588-024-01930-4

Yield of genetic association signals from genomes, exomes and imputation in the UK Biobank

In the format provided by the authors and unedited

Table of Contents

Supplementary Note 2

Survey of coding variation 2

Supplementary Figure 1. 3

Supplementary Figure 2. 4

Supplementary Figure 3. 5

Supplementary Figure 4 6

Supplementary Figure 5. 7

Supplementary Figure 6. 8

Supplementary Figure 7. 9

Supplementary Figure 8. 10

Supplementary Figure 9. 11

Supplementary Figure 10. 12

Supplementary Figure 11. 13

Supplementary Figure 12. 14

Supplementary Figure 13. 15

Supplementary Table 1 16

Supplementary Table 2 16

Supplementary Table 3 16

Supplementary Table 4 18

Supplementary Table 5 18

Supplementary Table 6 19

Supplementary Table 7 19

Supplementary Table 8 19

Supplementary Data 1. 20

Supplementary Data 2. 20

Supplementary Data 3. 20

List of Investigators from the Regeneron Genetics Center 20

References 21

Supplementary Note

Survey of coding variation

Before proceeding to genetic association analyses, we compared the number of coding variants detected by the WGS and WES+IMP approaches. We annotated variants by functional consequence with the ENSEMBL Variant Effect Predictor (McLaren et al., 2016) using the ENSEMBL 100 canonical transcript definitions (Cunningham et al., 2022). As expected, both approaches resulted in very similar numbers of coding variants per individual (WGS median: 19,905, IQR: 239; WES+IMP median: 19,948, IQR: 245). For both datasets, 48% of observed variants were singletons. For WGS, 75.3% variants are present in less than 5 individuals and similarly 74.7% of WES+IMP variants are present in less than 5 individuals. Overall, coding variants were distributed across 19,377 genes in the WGS data, and across 18,446 genes in the WES+IMP data set (among the genes in WES+IMP dataset, variants in 347 genes were outside the exome target regions and detected only through arrays and imputation).

The total number of coding variants captured by each approach was very similar (WGS 6,732,108 variants; WES+IMP 6,761,880 variants) with 6,544,263 observed in both WGS and WES+IMP. Among variants that were present in only the WES+IMP dataset, there were 126,319 missense variants – compared to 88,448 missense variants specific to the WGS data – the largest increase for a coding variant consequence. In contrast, the largest proportional gain was for variants that were present only in the WGS data for in-frame indels or predicted-loss-of-function (pLOF) variants – there were 9.3% more pLOFs and 23.5% more in-frame indels specific to the WGS data, but only 7.2% more pLOFs and 5.6% more in-frame indels specific to the WES+IMP data. Overall, 2.7% of coding variants were observed only in WGS and 3.1% of variants were observed only in WES+IMP. The coding variation was even more similar when limiting comparison to the target capture regions (Supplementary Table 4).

Supplementary Figure 1.

Flowchart of analytical UKB sample. The analysis includes individuals from the UK Biobank with WES, imputed array, and WGS data with all analytical datasets in the dotted box. The primary analytical dataset includes 149,195 individuals who have all data sources available (bold); secondary datasets include (a) 468,169 individuals with WES and imputed array data and (b) a subset of 47,545 individuals with WGS.

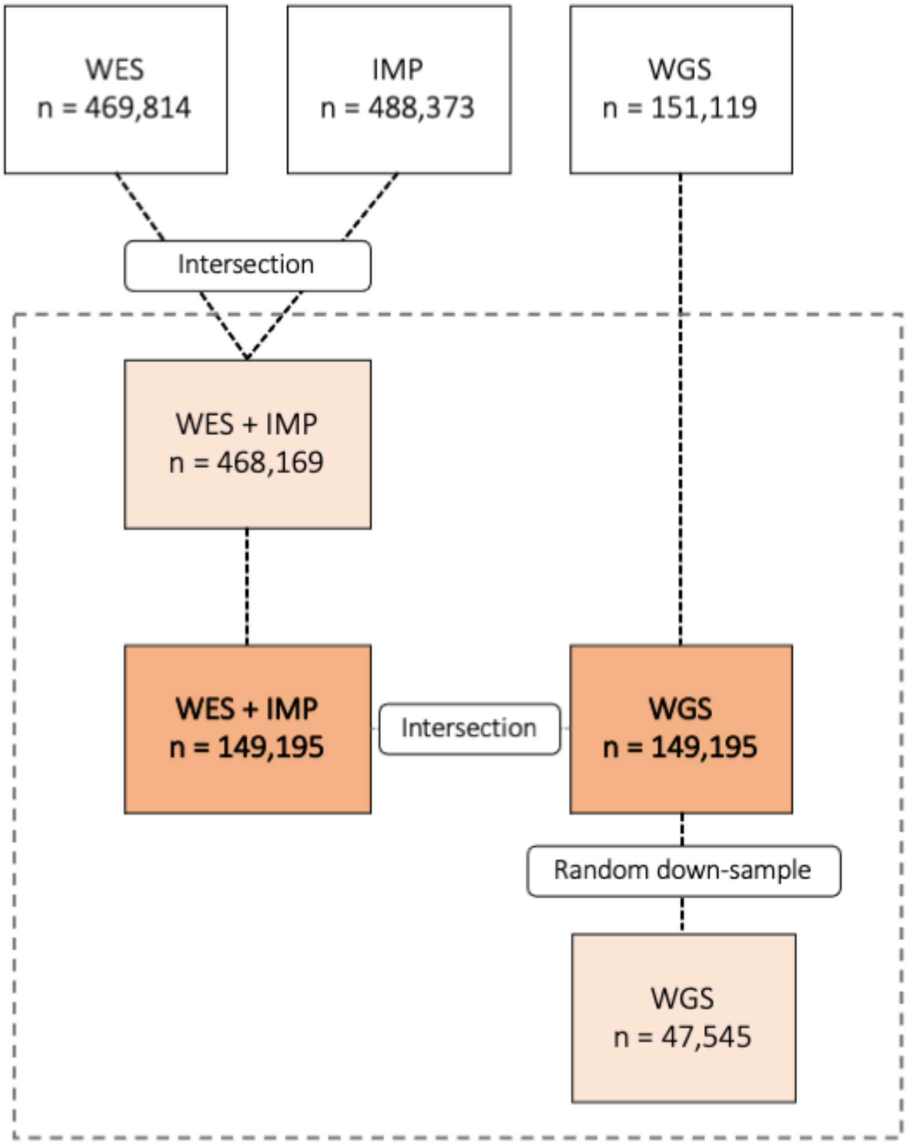

Supplementary Figure 2.

Flowchart of primary analyses. Analyses of the primary datasets (n=149,195) included performing single variant and gene-based association testing for WES, imputed array, and WGS data. The same tests were performed on the secondary datasets (n=468,169 and n=47,545).

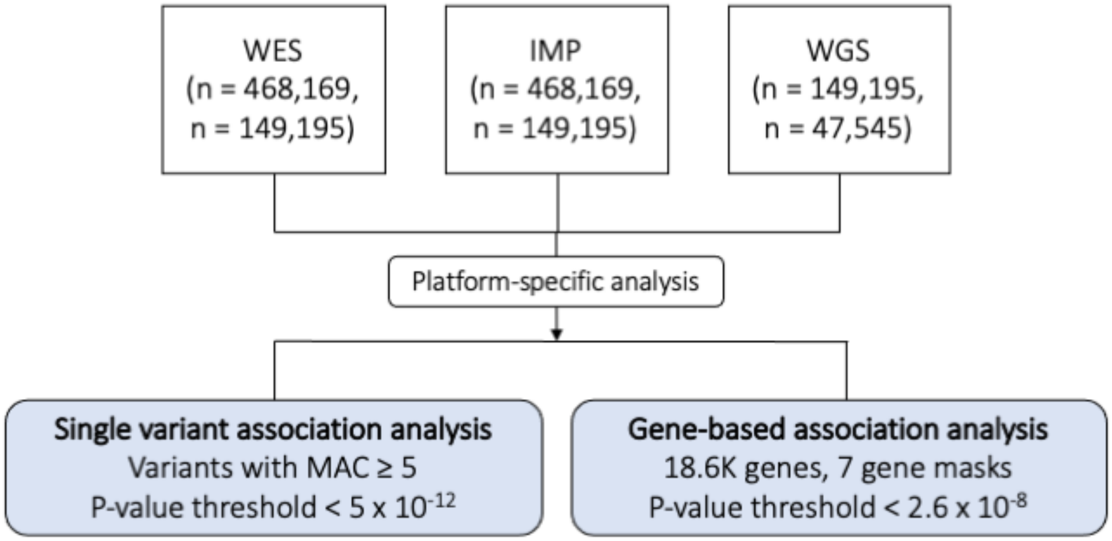

Supplementary Figure 3.

Survey of coding variation for WGS and WES+IMP. A comparison of the coding variation observed by the WES+IMP and WGS datasets stratified by functional consequence. In Panel A, the count of variants observed in each approach, in both approaches, and in only one approach is given; the percentage gains in approach-specific variants is also given. In Panel B, the variant count per individual (n=149,195) is given; the point provides the average and the error bars representing the corresponding standard errors.

A

Consequence	WGS (% Singleton)	WES+IMP (% Singleton)	Intersection	WGS only	WES+IMP only	% WGS only	% WES+IMP only
**Coding variants**	6,732,108 (48%)	6,761,880 (48%)	6,544,263	187,845	217,617	2.7	3.1
**Missense**	4,225,468 (49%)	4,263,339 (48%)	4,137,020	88,448	126,319	2.0	2.9
**Synonymous**	1,994,972 (44%)	2,012,866 (44%)	1,959,714	35,258	53,152	1.7	2.6
**In-frame indel**	87,080 (51%)	70,543 (45%)	65,383	21,697	5,160	23.5	5.6
**pLoF**	424,588 (60%)	415,132 (58%)	382,146	42,442	32,986	9.3	7.2
Frameshift	190,713 (63%)	182,447 (60%)	162,815	27,898	19,632	13.3	9.3
Stop gained	130,968 (56%)	132,452 (56%)	126,845	4,123	5,607	3.0	4.1
Start lost	12,058 (52%)	11,744 (51%)	11,374	684	370	5.5	3.0
Stop lost	4,599 (58%)	4,417 (57%)	4,184	415	233	8.6	4.8
Splice donor	49,170 (60%)	46,658 (59%)	43,151	6,019	3,507	11.4	6.7
Splice acceptor	37,080 (62%)	37,414 (61%)	33,777	3,303	3,637	8.1	8.9

B

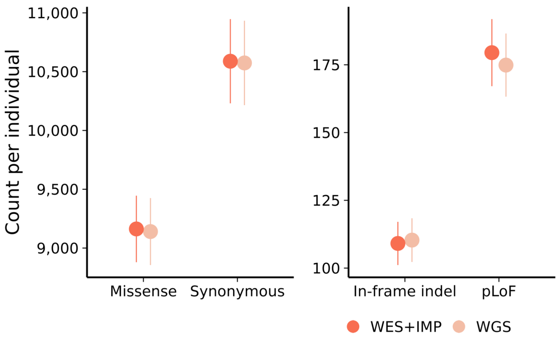

Supplementary Figure 4.

Flowchart of unified GENE_P test. Gene-based association analyses primarily focused on a single, unified p-value per gene. This gene-p p-value aggregates across multiple variant frequencies, masks (Supplementary Table 6), and set-based testing methods. The flowchart visualized how the single variants from a given gene are combined and tested to yield a single gene-level p-value, where gray arrows indicate aggregation by ACAT (Liu et al., 2019).

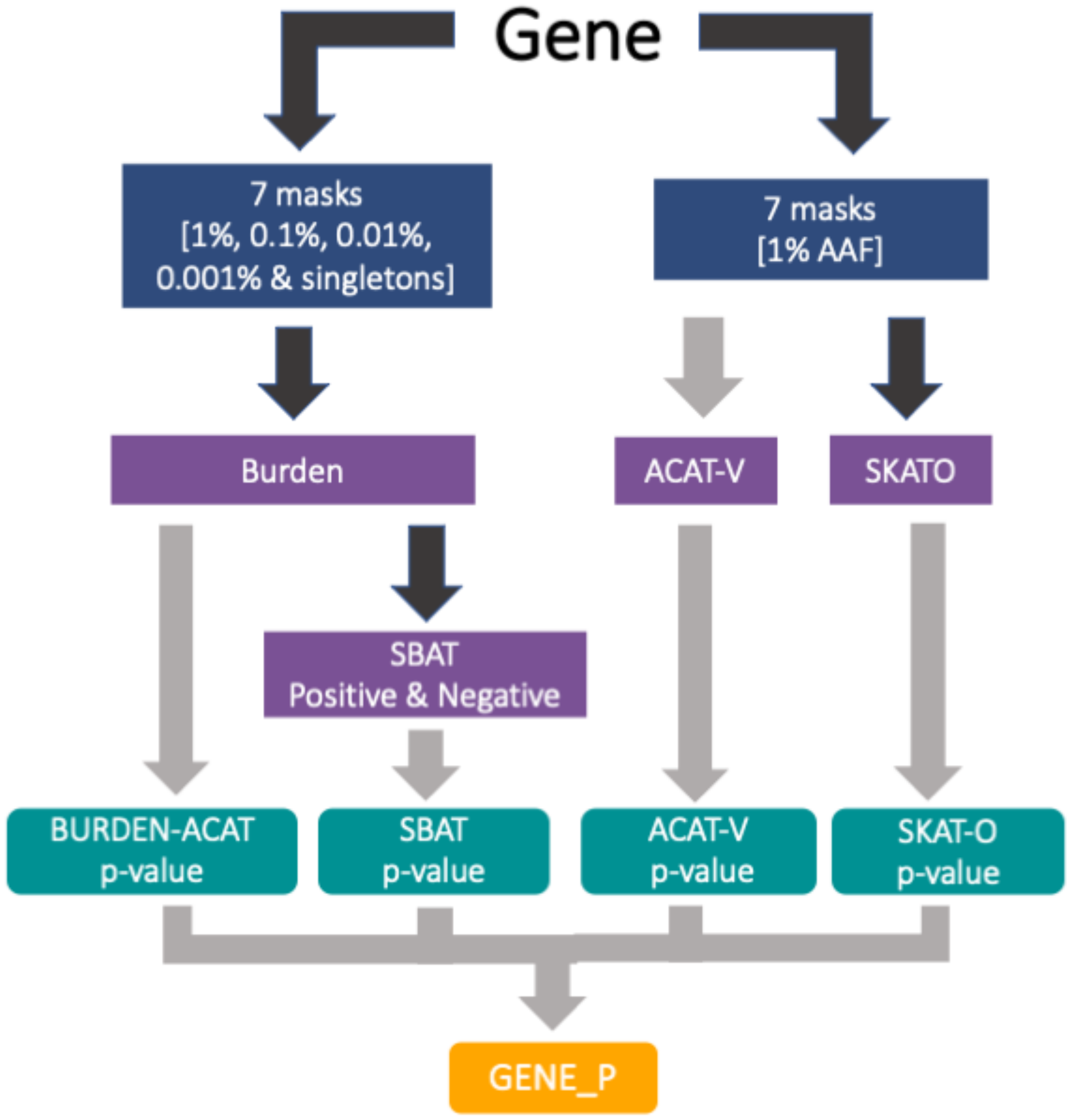

Supplementary Figure 5.

Summary of allele frequency, effect size, and p-value of all single variant association signals. Single variant a ssociations are grouped by the platform in which they were observed. Key features of the signals, AAF, effect siz e, and -log10(p-value), are plotted for each signal in all groups. The median value is plotted with box bounded b y 25th and 75th percentiles, with whiskers extending from the box to values within 1.5*Interquartile Range, and outlying values (minima/maxima) as points.

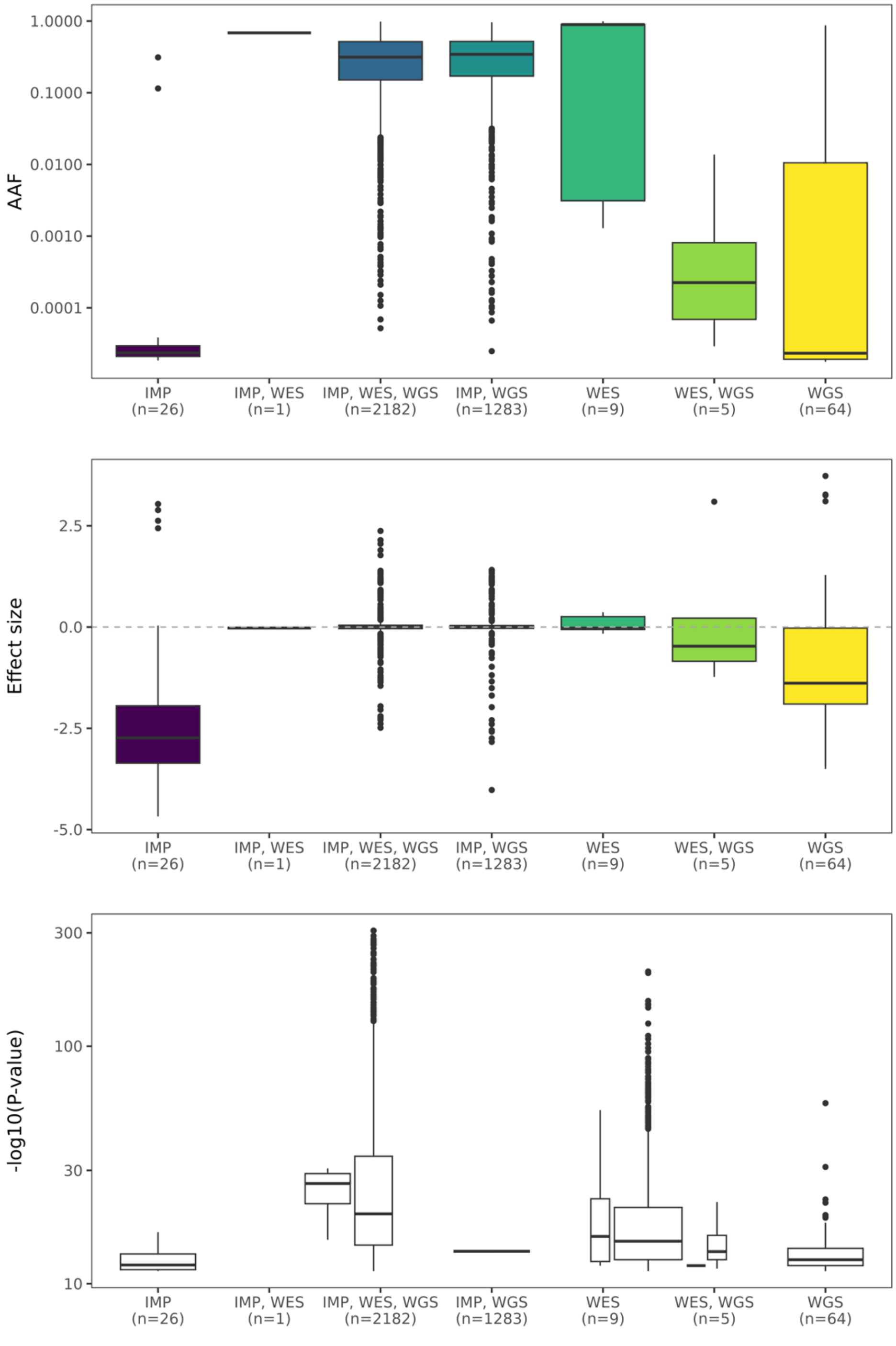

Supplementary Figure 6.

LocusZoom plot of lead single variant signals detected by all platforms. A 1Mb region centered around most significant single variant association that was supported across all platforms (WGS, WES, IMP, WGS – SV). This was defined as observing an association with a p-value within an order of magnitude of the threshold of significance within a 1Mb region of the index association. The association is shown for 12:21178615:T:C (rs4149056), associated with Total bilirubin and identified first from the WGS sequencing data with p-value 2.23e-307. This missense variant lies in an exon of gene *SLCO1B1*, and is commonly observed with AAF=0.15.

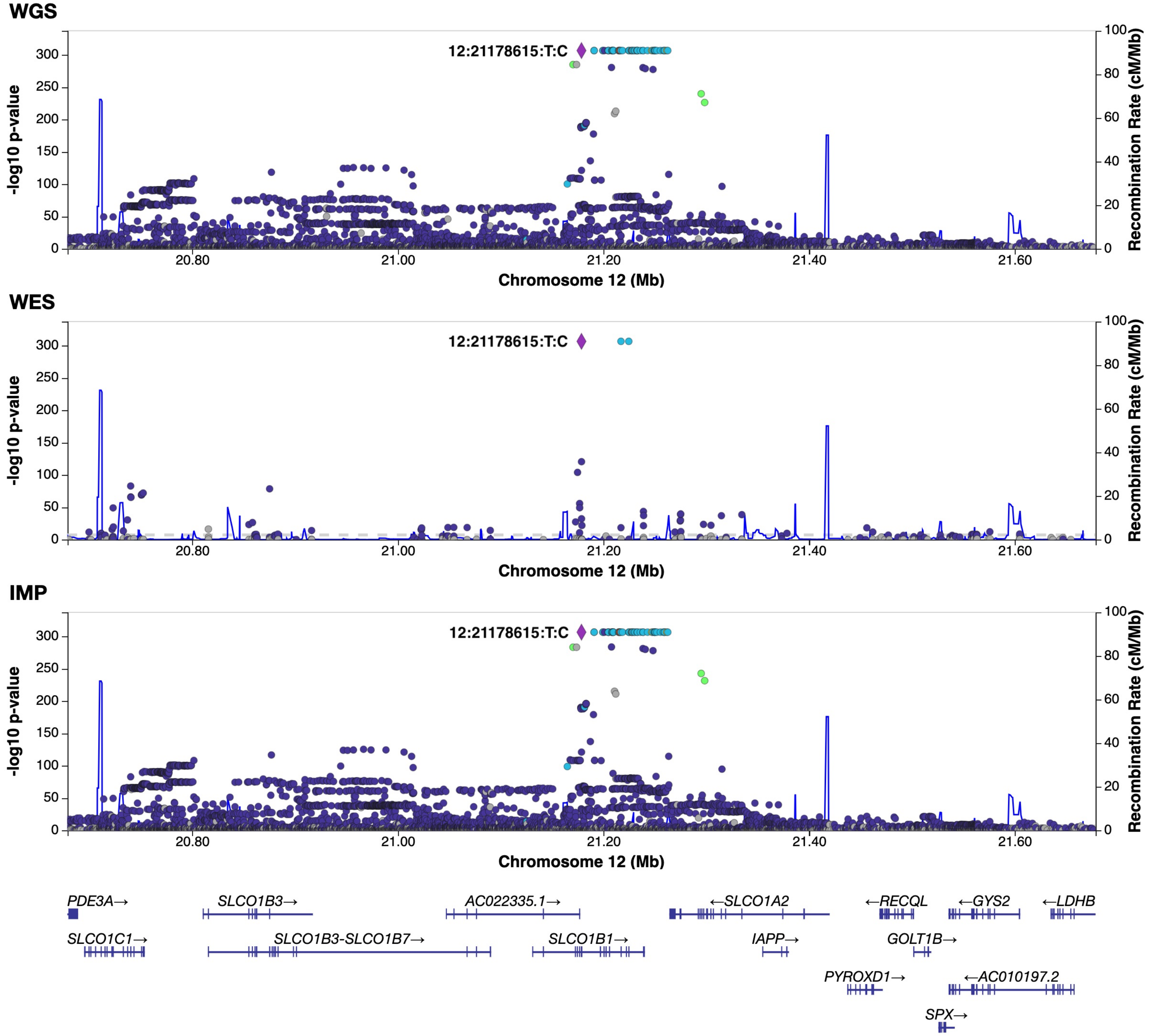

Supplementary Figure 7.

LocusZoom plots of single variant signals detected only by WGS. A 1Mb region centered on a peak single vari ant association signal observed only in WGS. This intronic variant, 4:23156769:C:T, is associated with forced vita l capacity with p-value 3.06e-12 and AAF=1.9e-5. It is supported by additional associated variants in WGS.

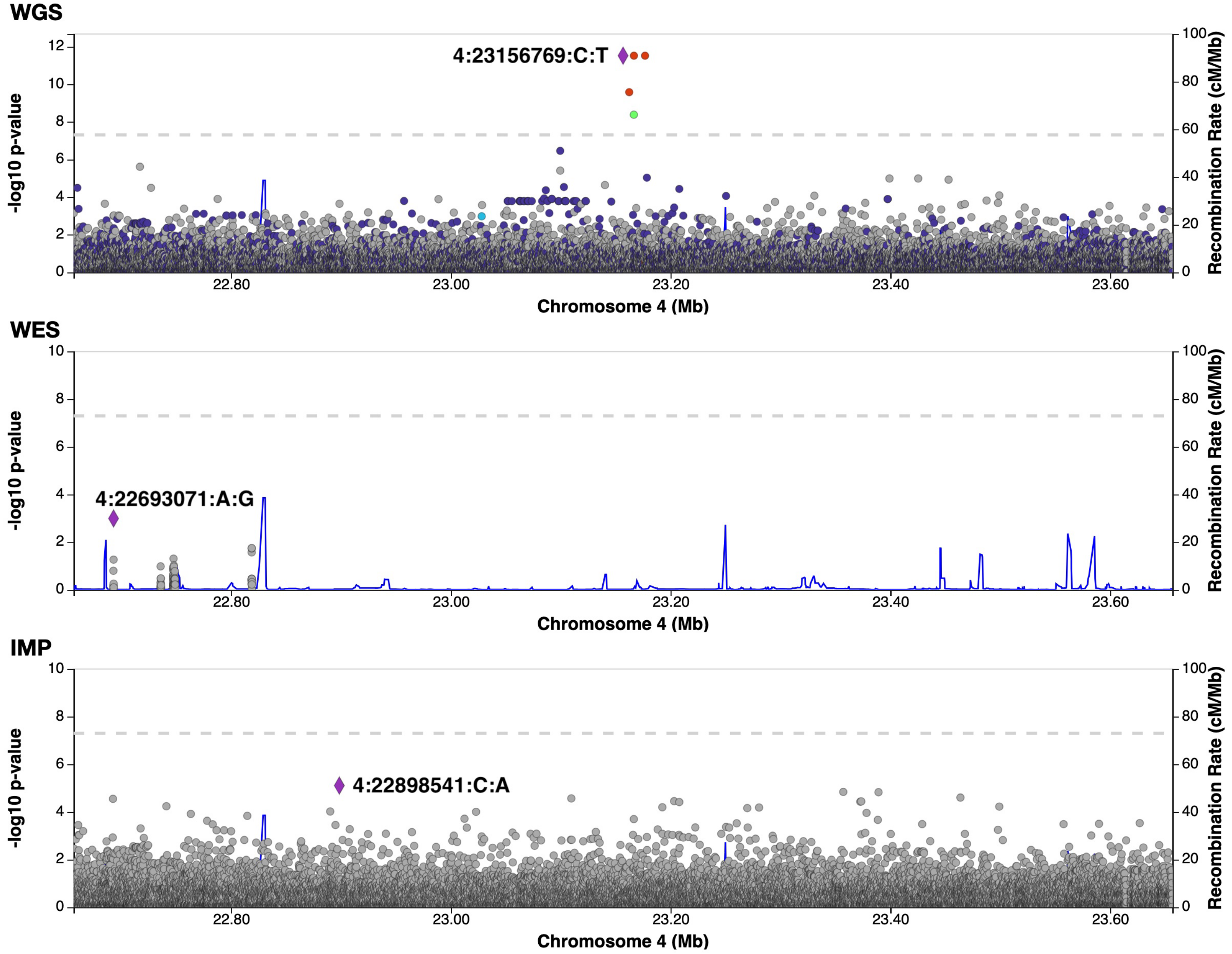

Supplementary Figure 8.

LocusZoom plots of single variant signals detected only by WES. A 1Mb region centered on a peak single variant association signal observed only in WES. Variant 14:33367284:AAAG:A is an intronic variant in gene *NPAS3*. It is associated with mean reticulocyte volume with p-value 3.99e-13 and AAF 0.0020. The most significant variant in the region in WES is neighboring with a p-value below the commonly recognized 5e-8 GWAS threshold. Similar signal is not observed in WGS and IMP.

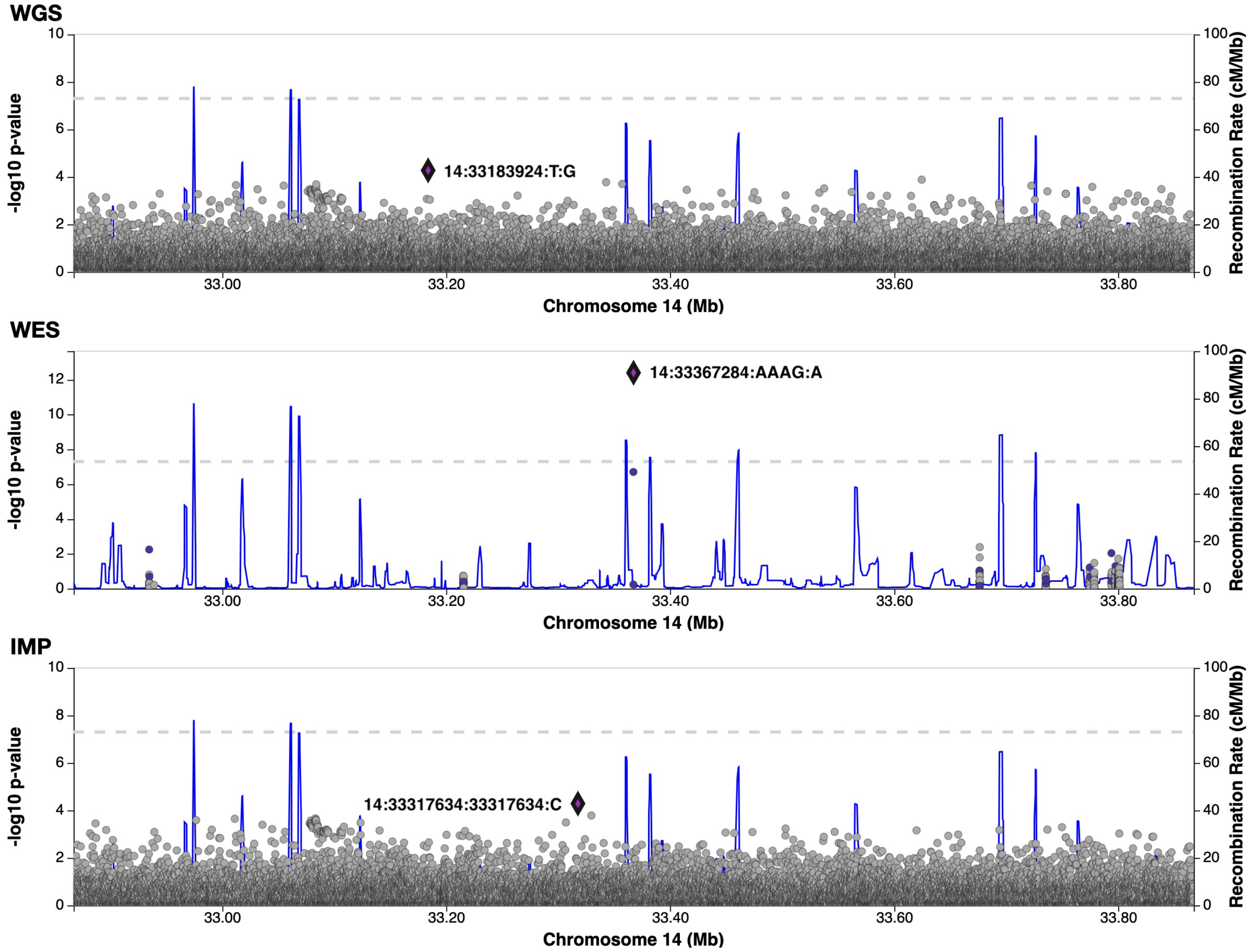

Supplementary Figure 9.

LocusZoom plots of single variant signals detected only by IMP. A 1Mb region centered on a peak single variant association signal observed only in IMP. This intergenic variant, 9:104216027:T:G, is associated with impedance of whole body with p-value 2.49e-12 and AAF=3.1e-5. It is supported by other variants in IMP.

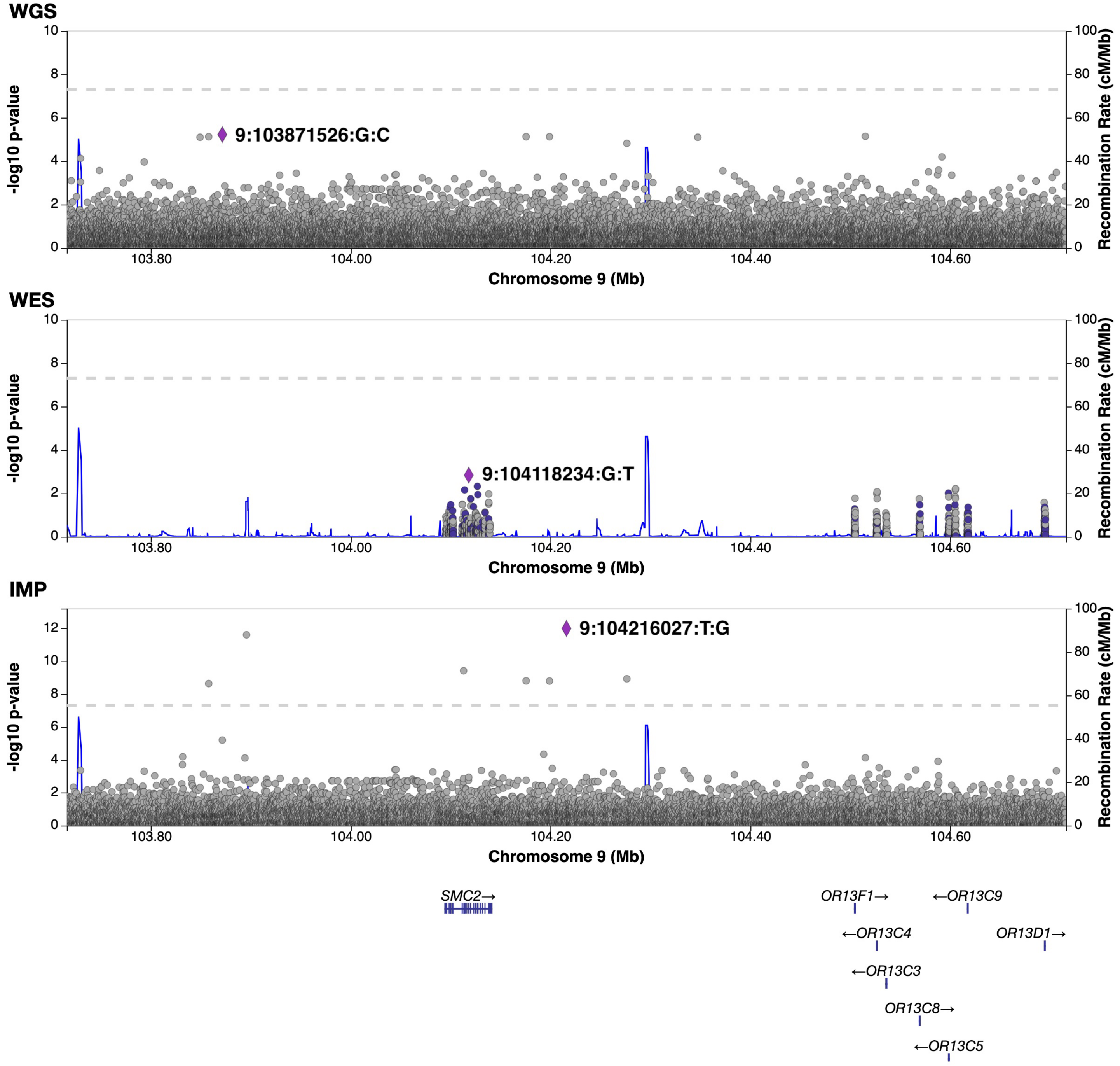

Supplementary Figure 10.

Summary of p-value of all gene-based association signals. Gene-based associations are grouped by the platform in which they were observed. The unified GENE_P p-value, incorporating multiple statistical tests and masks, are plotted for each signal in all groups. The median value is plotted with box bounded by 25th and 75th percentiles, with whiskers extending from the box to values within 1.5*Interquartile Range, and outlying values (minima/maxima) as points.

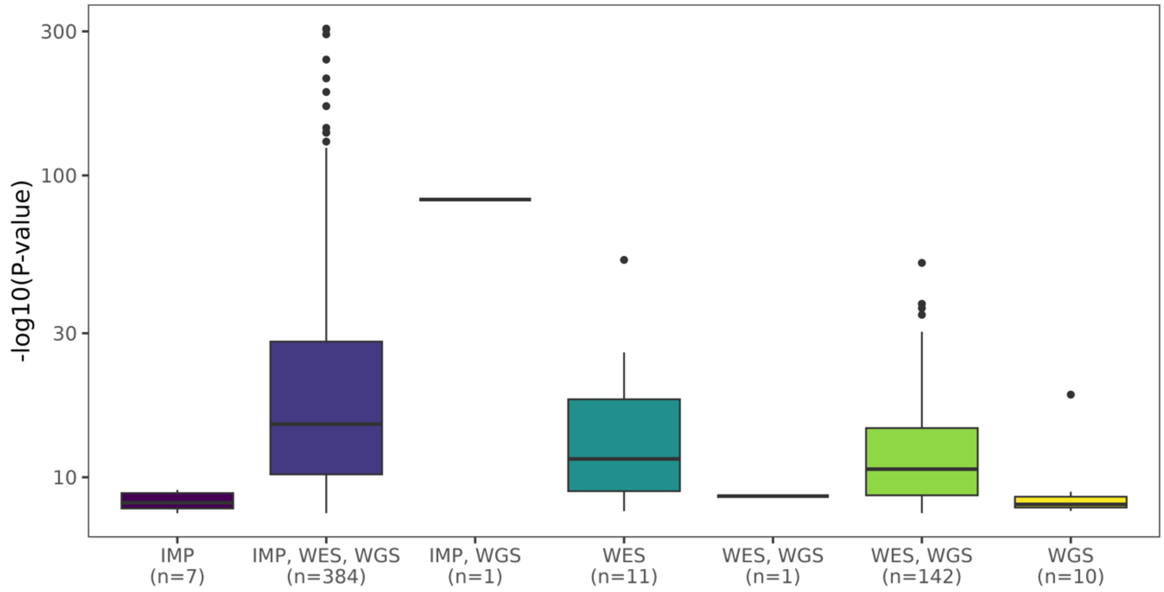

Supplementary Figure 11.

Comparison of GENE_P p-values for gene-based analyses between platforms. For each gene tested, the two-sided, unadjusted p-value between each pair of platforms is given for all tests and for those with $-\log_{10}(p - value)$.

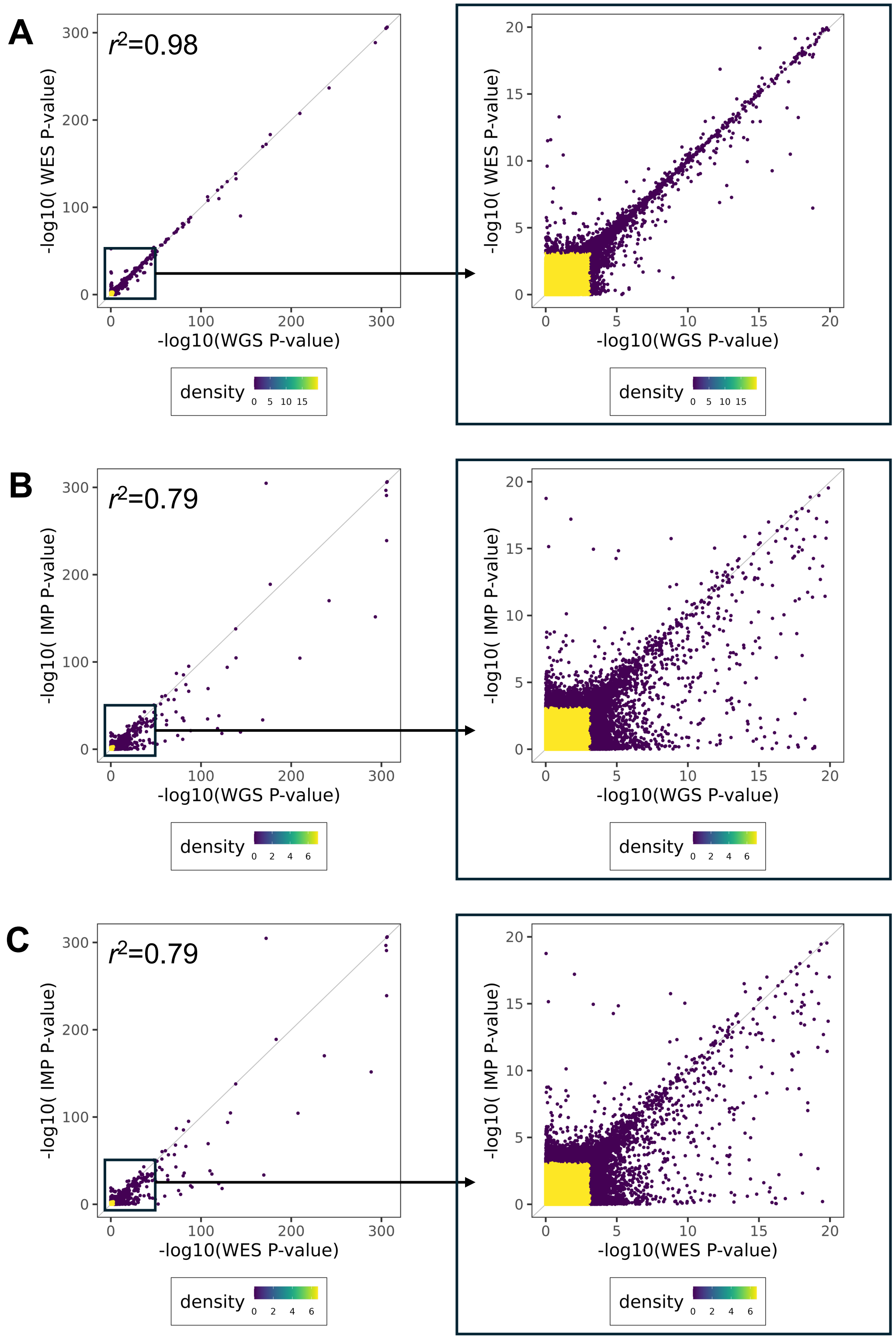

Supplementary Figure 12.

Evaluation of TOPMed-based imputation accuracy (R^2) across ancestry groups. Across allele frequency bins, where sufficient variation was observed, the imputation accuracy is given in comparison to the observed WGS.

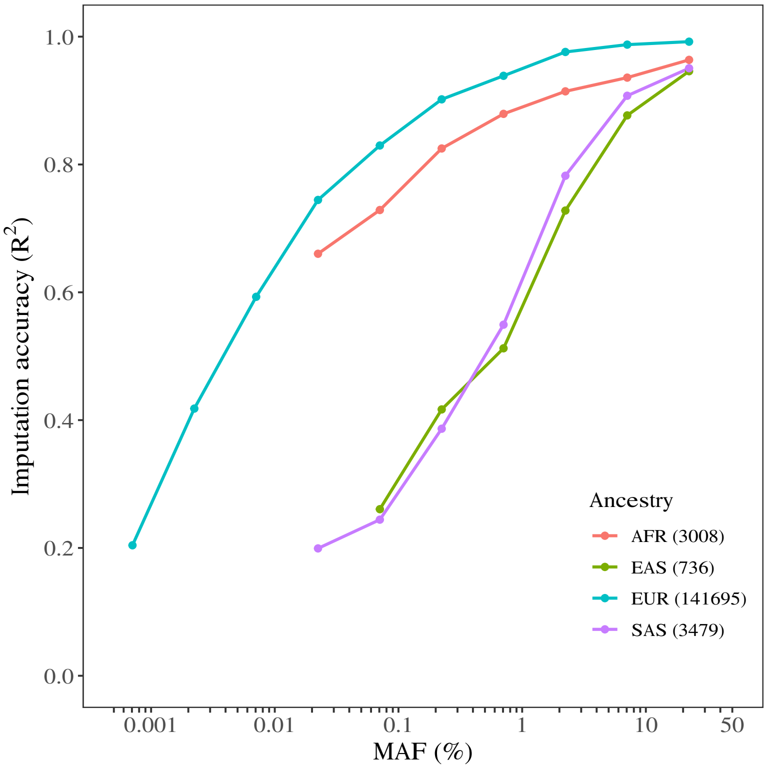

Supplementary Figure 13.

Comparison of TOPMed-based and UKB-based imputation accuracy (R^2). We imputed 1,000 white British individuals who had both WGS and array data. Imputation was based on (1) a reference panel generated from 200K UKB phased WGS samples and (2) a TOPMed reference panel, and compared. Across allele frequency bins, where sufficient variation was observed, the imputation accuracy is given for imputation based on a TOPMed reference panel and UKB 200K reference panel.

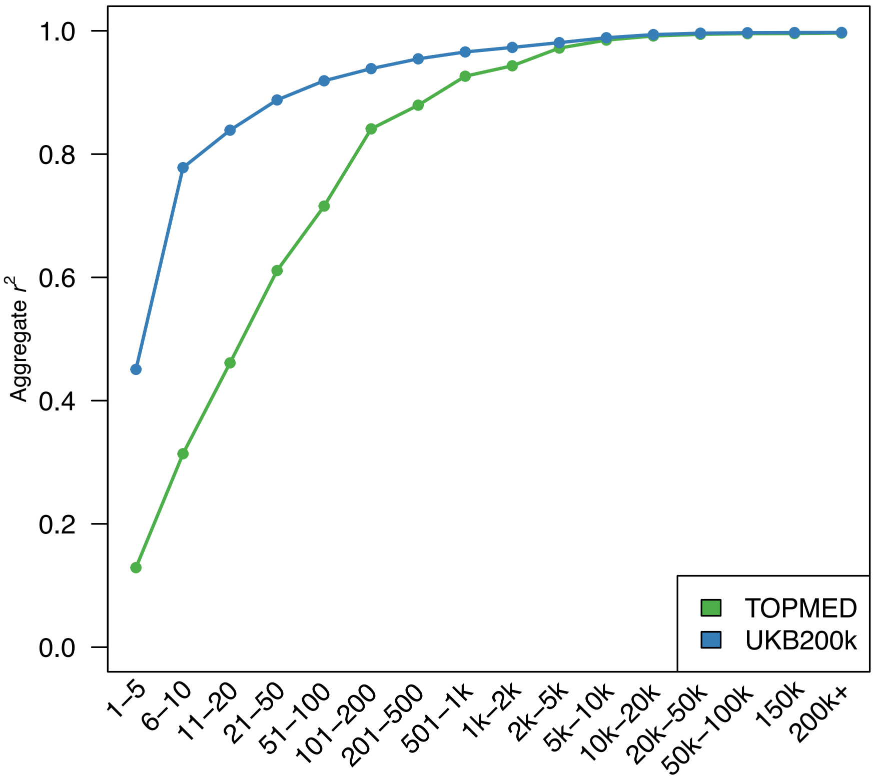

Supplementary Table 1.

Characteristics of the UKB data. Analyses included UKB data with three sample sizes, comprised of individuals with an assigned ancestry. Demographic features of these individuals are provided.

Sample size	Platforms analyzed	Sex - Female	Mean age (SD)	Ancestry	
47,545	WGS	26,384 (55.5%)	56.5 (8.1)	982	AFR
				97	AMR
				227	EAS
				45,093	EUR
				1,146	SAS
149,195	IMP, WES, WGS	82,210 (55.1%)	56.5 (8.1)	3,008	AFR
				277	AMR
				736	EAS
				141,695	EUR
				3,479	SAS
468,169	IMP, WES	253,697 (54.2%)	56.5 (8.1)	9,277	AFR
				856	AMR
				2,303	EAS
				445,544	EUR
				10,189	SAS

Supplementary Table 2.

Genotype concordance between the different approaches. For all autosomal variants that passed QC in each platform, after enforcing hard calls, we assessed the number of mean discordant calls per variant and the concordance across all variant calls.

Datasets	Number of variants compared	Mean number of discordant calls per variant	Concordance
WGS & WES	15,840,174	9.6	99.99%
WES & IMP	2,371,567	75.3	99.95%
WGS & IMP	89,809,894	72.2	99.95%

Supplementary Table 3.

Number of canonical coding variants in WGS and WES+IMP datasets. Count of variants for each coding consequence, stratified by frequency. Variants were annotated with VEP and genes were defined by Ensembl v100.

Consequence	Frequency	WGS	WES+IMP	Union	Intersection	WGS only	WES+IMP only
Missense	Singleton (AAC=1)	2,055,463	2,056,701	2,113,288	1,998,876	56,587	57,825
	AAC>1 & AAF<=0.0001	1,935,023	1,968,371	1,999,707	1,903,687	31,336	64,684
	AAF>0.001 & AAF<=0.01	208,238	211,273	211,740	207,771	467	3,502
	AAF>0.01	26,744	26,994	27,052	26,686	58	308
Stop gained	Singleton (AAC=1)	73,515	73,664	76,502	70,677	2,838	2,987
	AAC>1 & AAF<=0.0001	53,823	55,045	56,312	52,556	1,267	2,489
	AAF>0.001 & AAF<=0.01	3,429	3,537	3,554	3,412	17	125
	AAC>1 & AAF<=0.0001	53,823	55,045	56,312	52,556	1,267	2,489
	AAF>0.01	201	206	207	200	1	6
Synonymous	Singleton (AAC=1)	884,307	886,016	907,833	862,490	21,817	23,526
	AAC>1 & AAF<=0.0001	943,930	958,636	971,785	930,781	13,149	27,855

	AAF>0.001 & AAF<=0.01	138,548	139,882	140,132	138,298	250	1,584
	AAF>0.01	28,187	28,332	28,374	28,145	42	187
In-frame indel	Singleton (AAC=1)	44,268	31,537	46,642	29,163	15,105	2,374
	AAC>1 & AAF<=0.0001	38,109	34,143	40,573	31,679	6,430	2,464
	AAF>0.001 & AAF<=0.01	4,311	4,450	4,599	4,162	149	288
	AAF>0.01	392	413	426	379	13	34
Frameshift	Singleton (AAC=1)	120,430	108,817	130,794	98,453	21,977	10,364
	AAC>1 & AAF<=0.0001	65,982	68,518	74,362	60,138	5,844	8,380
	AAF>0.001 & AAF<=0.01	4,065	4,813	4,878	4,000	65	813
	AAF>0.01	236	299	311	224	12	75
Splice donor	Singleton (AAC=1)	27,177	27,000	29,192	24,985	2,192	2,015
	AAC>1 & AAF<=0.0001	16,549	17,209	17,875	15,883	666	1,326
	AAF>0.001 & AAF<=0.01	1,097	1,143	1,155	1,085	12	58
	AAF>0.01	63	67	70	60	3	7
Splice acceptor	Singleton (AAC=1)	21,344	21,693	23,321	19,716	1,628	1,977
	AAC>1 & AAF<=0.0001	11,993	12,814	13,320	11,487	506	1,327
	AAF>0.001 & AAF<=0.01	721	749	752	718	3	31
	AAF>0.01	39	41	41	39	0	2
Start lost	Singleton (AAC=1)	6,250	5,968	6,418	5,800	450	168
	AAC>1 & AAF<=0.0001	5,351	5,307	5,537	5,121	230	186
	AAF>0.001 & AAF<=0.01	421	432	436	417	4	15
	AAF>0.01	36	37	37	36	0	1
Stop lost	Singleton (AAC=1)	2,678	2,509	2,810	2,377	301	132
	AAC>1 & AAF<=0.0001	1,751	1,736	1,847	1,640	111	96
	AAF>0.001 & AAF<=0.01	153	155	158	150	3	5
	AAF>0.01	17	17	17	17	0	0
5’ UTR splice acceptor	Singleton (AAC=1)	1,559	1,113	1,741	931	628	182
	AAC>1 & AAF<=0.0001	1,066	811	1,171	706	360	105
	AAF>0.001 & AAF<=0.01	94	98	99	93	1	5
	AAF>0.01	10	11	11	10	0	1
3’ UTR splice acceptor	Singleton (AAC=1)	134	23	136	21	113	2
	AAC>1 & AAF<=0.0001	103	42	106	39	64	3
	AAF>0.001 & AAF<=0.01	16	18	18	16	0	2
	AAF>0.01	1	1	1	1	0	0
5’ UTR splice donor	Singleton (AAC=1)	2,119	284	2,139	264	1,855	20
	AAC>1 & AAF<=0.0001	1,655	476	1,699	432	1,223	44

	AAF>0.001 & AAF<=0.01	155	152	158	149	6	3
	AAF>0.01	25	25	25	25	0	0
3’ UTR splice donor	Singleton (AAC=1)	178	153	199	132	46	21
	AAC>1 & AAF<=0.0001	124	121	136	109	15	12
	AAF>0.001 & AAF<=0.01	20	21	21	20	0	1
	AAF>0.01	8	7	8	7	1	0

Supplementary Table 4.

Number of canonical coding variants in target capture regions for WGS and WES+IMP datasets. Count of variants for each coding consequence when limiting to variants within the WES targeted capture regions. Variants were annotated with VEP and genes were defined by Ensembl v100. pLoFs included frameshift, splice donor, splice acceptor, stop gained, stop lost, and start lost variants.

Consequence	WGS	WES+IMP	Union	Intersection	WGS only	WES+IMP only
Missense	4155578	4244803	4279583	4120798	34780	124005
Synonymous	1964215	2003872	2016349	1951738	12477	52134
In-frame indel	84619	69754	89655	64718	19901	5036
pLoF	358211	356825	385531	329505	28706	27320

Supplementary Table 5.

Single variant signal consequences by platforms with association observed. Count of variants for each consequence with an observed trait association, given by the platforms in which the signal is observed. Variants were annotated with VEP and genes were defined by Ensembl v100.

Consequence	IMP	IMP, WES	IMP, WES, WGS	IMP, WGS	WES	WES, WGS	WGS
3' UTR	0	0	75	27	0	0	1
5' UTR	0	0	27	9	0	0	0
Downstream	1	0	108	23	0	0	2
Frameshift	0	0	5	0	0	1	0
In-frame indel	0	0	6	0	0	0	0
Intergenic	17	0	422	691	0	0	38
Intronic	8	1	1,014	467	7	2	22
Missense	0	0	281	0	1	1	0
Splice donor	0	0	2	0	0	1	0
Splice region	0	0	6	0	0	0	0
Stop gained	0	0	10	0	0	0	0
Synonymous	0	0	28	0	0	0	0
Upstream	0	0	198	66	1	0	1

Supplementary Table 6.

Gene burden mask definitions. For gene-based testing, variants were grouped into seven different masks by variant consequence. The variants were annotated with VEP and Ensembl 100, and aggregated into masks for tests in Regenie.

Mask	Variant consequences included
pLoF	stop_gained, stop_gain, frameshift, splice_donor, splice_acceptor
pLoF_missense_5	stop_gained, stop_gain, frameshift, splice_donor, splice_acceptor, start_lost, stop_lost, missense(5/5)
pLoF_missense_1	stop_gained, stop_gain, frameshift, splice_donor, splice_acceptor, start_lost, stop_lost, missense(5/5), missense(>=1/5), UTR_splice_donor, 5_prime_UTR_splice_donor, 3_prime_UTR_splice_donor, UTR_splice_acceptor, 5_prime_UTR_splice_acceptor, 3_prime_UTR_splice_acceptor
pLoF_missense_0	stop_gained, stop_gain, frameshift, splice_donor, splice_acceptor, start_lost, stop_lost, missense(5/5), missense(>=1/5), missense(0/5), UTR_splice_donor, 5_prime_UTR_splice_donor, 3_prime_UTR_splice_donor, UTR_splice_acceptor, 5_prime_UTR_splice_acceptor, 3_prime_UTR_splice_acceptor
missense_5	start_lost, stop_lost, missense(5/5)
missense_1	start_lost, stop_lost, missense(5/5), missense(>=1/5)
missense_0	start_lost, stop_lost, missense(5/5), missense(>=1/5), missense(0/5)

Supplementary Table 7.

Single and rare variant signals when considering alternative significance thresholds. For single variant testing, we summarize results for the association analysis using our main analysis significance threshold of $P=5 \times 10^{-12}$ and under a relaxed significance threshold of $P=5 \times 10^{-10}$. For gene-based testing, we summarize the results of our analysis using our main analysis significant threshold of $P=2.6 \times 10^{-8}$ and a relaxed significant threshold of $P=2.6 \times 10^{-6}$.

	Single variant analysis		Gene-based analysis	
	Number of signals, main analysis	Number of signals, relaxed threshold	Number of signals, main analysis	Number of signals, relaxed threshold
Shared	3,470	4,889	528	859
WGS only	64	241	10	21
WES+IMP only	36	167	18	38

Supplementary Table 8.

Single variant signals detected only by structural variant analyses. We analyzed structural variants from the WGS data using the single variant approach, and then jointly performed peak finding with the WGS, WES, and imputed data (significance threshold of $P=5 \times 10^{-12}$). The signals exclusive to structural variants are summarized.

Trait	Structural variant	P-value
30080_Platelet_count_inst_mean__RINT	chr17:25233296:N:<DUP:SVSIZE=343:AGGREGATED>:SV	5.24E-13
30260_Mean_reticulocyte_volume_inst_mean__RINT	chr17:25828349:N:<DUP:SVSIZE=672:AGGREGATED>:SV	5.71E-29
30260_Mean_reticulocyte_volume_inst_mean__RINT	chr1:113416208:N:<DEL:SVSIZE=88:AGGREGATED>:SV	8.23E-14
30260_Mean_reticulocyte_volume_inst_mean__RINT	chr1:118319009:N:<DEL:SVSIZE=63:AGGREGATED>:SV	9.64E-20
30260_Mean_reticulocyte_volume_inst_mean__RINT	chr1:194098949:N:<DEL:SVSIZE=299:AGGREGATED>:SV	8.58E-19
30260_Mean_reticulocyte_volume_inst_mean__RINT	chr1:197164767:N:<DEL:SVSIZE=59:AGGREGATED>:SV	6.69E-14
30260_Mean_reticulocyte_volume_inst_mean__RINT	chr1:232368256:N:<DEL:SVSIZE=225:AGGREGATED>:SV	4.92E-19
30260_Mean_reticulocyte_volume_inst_mean__RINT	chr1:91169079:N:<DEL:SVSIZE=126:AGGREGATED>:SV	3.39E-15
30260_Mean_reticulocyte_volume_inst_mean__RINT	chr1:97323337:N:<DEL:SVSIZE=119:AGGREGATED>:SV	4.26E-20
30260_Mean_reticulocyte_volume_inst_mean__RINT	chr5:164328528:N:<DEL:SVSIZE=606:AGGREGATED>:SV	1.54E-13
30810_Phosphate_inst_mean__RINT	chr17:25828349:N:<DUP:SVSIZE=672:AGGREGATED>:SV	5.98E-18
30810_Phosphate_inst_mean__RINT	chr1:118319009:N:<DEL:SVSIZE=63:AGGREGATED>:SV	2.23E-13

30810_Phosphate_inst_mean__RINT	chr1:97323337:N:<DEL:SVSIZE=119:AGGREGATED>:SV	1.02E-18
30870_Triglycerides_inst_mean__RINT	chr3:172556424:N:<DEL:SVSIZE=2317:AGGREGATED>:SV	2.38E-12
30880_Urate_inst_mean__RINT	chr1:124976466:N:<DEL:SVSIZE=2688:AGGREGATED>:SV	1.93E-25
3581_Age_at_menopause_last_menstrual_period_inst_mean__RINT	chr19:3939254:N:<DEL:SVSIZE=754:AGGREGATED>:SV	1.24E-12

Supplementary Data 1.

Significant single variant tests.

Attached.

Supplementary Data 2.

Significant gene-based tests.

Attached.

Supplementary Data 3.

List of 100 traits used in association analysis.

Attached.

List of Investigators from the Regeneron Genetics Center

RGC Management & Leadership Team

Aris Baras[1], Gonçalo R. Abecasis[1], Adolfo Ferrando[1], Michael Cantor[1], Giovanni Coppola[1], Andrew Deubler[1], Aris Economides[1], Luca A. Lotta[1], John D. Overton[1], Jeffrey G. Reid[1], Alan Shuldiner[1], Katherine Siminovitch[1], Jason Portnoy[1], Marcus B. Jones[1], Lyndon Mitnaul[1], Alison Fenney[1], Jonathan Marchini[1], Manuel Allen Revez Ferreira[1], Maya Ghoussaini[1], Mona Nafde[1], William J. Salerno[1]

Sequencing & Lab Operations

John D. Overton[1], Christina Beechert[1], Erin D. Brian[1], Laura M. Cremona[1], Hang Du[1], Caitlin Forsythe[1], Zhenhua Gu[1], Kristy Guevara[1], Michael Lattari[1], Alexander Lopez[1], Kia Manoochehri[1], Prathyusha Challa[1], Manasi Pradhan[1], Raymond Reynoso[1], Ricardo Schiavo[1], Maria Sotiropoulos Padilla[1], Chenggu Wang[1], Sarah E. Wolf[1]

Clinical Informatics

Michael Cantor[1], Amelia Averitt[1], Nilanjana Banerjee[1], Dadong Li[1], Sameer Malhotra[1], Justin Mower[1], Mudasar Sarwar[1], Deepika Sharma[1], Jeffrey C. Staples[1], Sean Yu[1], Aaron Zhang[1], Muhammad Aqeel[1]

Genome Informatics & Data Engineering

Jeffrey G. Reid[1], Mona Nafde[1], George Mitra[1], Sujit Gokhale[1], Krishna Pawan Punuru[1], Sanjay Sreeram[1], Gisu Eom[1], Sujit Gokhale[1], Benjamin Sultan[1], Rouel Lanche[1], Vrushali Mahajan[1], Eliot Austin[1], Sean O'Keeffe[1], Razvan Panea[1], Ayesha Rasool[1], William J. Salerno[1], Xiaodong Bai[1], Lance Zhang[1], Boris Boutkov[1], Evan Edelstein[1], Alexander Gorovits[1], Ju Guan[1], Lukas Habegger[1], Alicia Hawes[1], Olga Krasheninina[1], Samantha Zarate[1], Adam J. Mansfield[1], Evan K. Maxwell[1], Suganthi Balasubramanian[1], Suying Bao[1], Kathie Sun[1], Chuanyi Zhang[1]

Analytical Genetics and Data Science

Gonçalo R. Abecasis[1], Manuel Allen Revez Ferreira[1], Joshua Backman[1], Kathy Burch[1], Adrian Campos[1], Lei Chen[1], Sam Choi[1], Amy Damask[1], Liron Ganel[1], Sheila M. Gaynor[1], Benjamin Geraghty[1], Arkopravo Ghosh[1], Salvador Romero Martinez[1], Christopher Gillies[1], Lauren Gurski[1], Joseph Herman[1], Eric Jorgenson[1], Tyler Joseph[1], Michael Kessler[1], Jack Kosmicki[1], Nan Lin[1], Adam Locke[1], Priyanka Nakka[1], Jonathan Marchini[1], Karl Landheer[1], Olivier Delaneau[1], Maya Ghoussaini[1], Anthony Marcketta[1], Joelle Mbatchou[1], Arden Moscati[1], Aditeya Pandey[1], Anita Pandit[1], Charles Paulding[1],

Jonathan Ross[1], Carlo Sidore[1], Eli Stahl[1], Maria Suciu[1], Timothy A. Thornton[1], Peter VandeHaar[1], Sailaja Vedantam[1], Scott Vrieze[1], Jingning Zhang[1], Rujin Wang[1], Kuan-Han Wu[1], Bin Ye[1], Blair Zhang[1], Andrey Ziyatdinov[1], Yuxin Zou[1], Olivier Delaneau[1], Maya Ghoussaini[1], Jingning Zhang[1], Kyoko Watanabe[1], Mira Tang[1], Frank Wendt[1]

Therapeutic Area Genetics
Adolfo Ferrando[1], Giovanni Coppola[1], Luca A. Lotta[1], Alan Shuldiner[1], Katherine Siminovitch[1], Brian Hobbs[1], Jon Silver[1], William Palmer[1], Rita Guerreiro[1], Amit Joshi[1], Antoine Baldassari[1], Cristen Willer[1], Sarah Graham[1], Ernst Mayerhofer[1], Mary Haas[1], Niek Verweij[1], George Hindy[1], Jonas Bovijn[1], Tanima De[1], Parsa Akbari[1], Luanluan Sun[1], Olukayode Sosina[1], Arthur Gilly[1], Peter Dornbos[1], Juan Rodriguez-Flores[1], Moeen Riaz[1], Manav Kapoor[1], Gannie Tzoneva[1], Momodou W. Jallow[1], Anna Alkelai[1], Giovanni Coppola[1], Ariane Ayer[1], Veera Rajagopal[1], Sahar Gelfman[1], Vijay Kumar[1], Jacqueline Otto[1], Neelroop Parikshak[1], Aysegul Guvenek[1], Jose Bras[1], Silvia Alvarez[1], Jessie Brown[1], Jing He[1], Hossein Khiabanian[1], Joana Revez[1], Kimberly Skead[1], Valentina Zavala[1], Jae Soon Sul[1]

Research Program Management & Strategic Initiatives
Marcus B. Jones[1], Esteban Chen[1], Michelle G. LeBlanc[1], Jason Mighty[1], Jennifer Rico-Varela[1], Nirupama Nishtala[1], Nadia Rana[1], Jaimee Hernandez[1]

Senior Partnerships & Business Operations
Alison Fenney[1], Randi Schwartz[1], Jody Hankins[1], Samuel Hart[1]

Business Operations & Administrative Coordinators
Ann Perez-Beals[1], Gina Solari[1], Johannie Rivera-Picart[1], Michelle Pagan[1], Sunilbe Siceron[1]

[1]Regeneron Genetics Center, Tarrytown, NY, USA.

References

Cunningham, F., Allen, J. E., Allen, J., Alvarez-Jarreta, J., Amode, M. R., Armean, I. M., Austine-Orimoloye, O., Azov, A. G., Barnes, I., & Bennett, R. (2022). Ensembl 2022. *Nucleic acids research*, *50*(D1), D988-D995.

Liu, Y., Chen, S., Li, Z., Morrison, A. C., Boerwinkle, E., & Lin, X. (2019). ACAT: a fast and powerful p value combination method for rare-variant analysis in sequencing studies. *The American Journal of Human Genetics*, *104*(3), 410-421.

McLaren, W., Gil, L., Hunt, S. E., Riat, H. S., Ritchie, G. R., Thormann, A., Flicek, P., & Cunningham, F. (2016). The ensembl variant effect predictor. *Genome biology*, *17*(1), 1-14.
