# Supplementary material for: Yield of genetic association signals from genomes, exomes and imputation in the UK Biobank
Source: Nat Genet. 2024 Sep 25;56(11):2345–51. doi: 10.1038/s41588-024-01930-4 (PMC11549045; doi:10.1038/s41588-024-01930-4)
Supplement: Supplementary file 2 — Reporting Summary [file 41588_2024_1930_MOESM2_ESM.pdf]

Corresponding author(s): Timothy A. Thornton

Last updated by author(s): August 7, 2024

Reporting Summary

Nature Portfolio wishes to improve the reproducibility of the work that we publish. This form provides structure for consistency and transparency in reporting. For further information on Nature Portfolio policies, see our Editorial Policies and the Editorial Policy Checklist.

Statistics

For all statistical analyses, confirm that the following items are present in the figure legend, table legend, main text, or Methods section.

n/a	Confirmed	
☐	☒	The exact sample size (*n*) for each experimental group/condition, given as a discrete number and unit of measurement
☐	☒	A statement on whether measurements were taken from distinct samples or whether the same sample was measured repeatedly
☐	☒	The statistical test(s) used AND whether they are one- or two-sided<br>*Only common tests should be described solely by name; describe more complex techniques in the Methods section.*
☐	☒	A description of all covariates tested
☐	☒	A description of any assumptions or corrections, such as tests of normality and adjustment for multiple comparisons
☐	☒	A full description of the statistical parameters including central tendency (e.g. means) or other basic estimates (e.g. regression coefficient) AND variation (e.g. standard deviation) or associated estimates of uncertainty (e.g. confidence intervals)
☐	☒	For null hypothesis testing, the test statistic (e.g. *F*, *t*, *r*) with confidence intervals, effect sizes, degrees of freedom and *P* value noted<br>*Give P values as exact values whenever suitable.*
☒	☐	For Bayesian analysis, information on the choice of priors and Markov chain Monte Carlo settings
☐	☒	For hierarchical and complex designs, identification of the appropriate level for tests and full reporting of outcomes
☐	☒	Estimates of effect sizes (e.g. Cohen's *d*, Pearson's *r*), indicating how they were calculated

Our web collection on statistics for biologists contains articles on many of the points above.

Software and code

Policy information about availability of computer code

Data collection	No software was used for data collection.
Data analysis	All genetic association analyses were performed using REGENIE (v3.1.1): https://rgcgithub.github.io/regenie/. Data was prepared using GraphTyper (v2.7.1): https://github.com/DecodeGenetics/graphtyper and Genome Analysis Toolkit (v.4.0.12): https://gatk.broadinstitute.org/hc/en-us. Code to reproduce analyses is available at GitHub (github.com/rgcgithub/ukb_genetic_association_yield).

For manuscripts utilizing custom algorithms or software that are central to the research but not yet described in published literature, software must be made available to editors and reviewers. We strongly encourage code deposition in a community repository (e.g. GitHub). See the Nature Portfolio guidelines for submitting code & software for further information.

Data

Policy information about availability of data

All manuscripts must include a data availability statement. This statement should provide the following information, where applicable:

- Accession codes, unique identifiers, or web links for publicly available datasets
- A description of any restrictions on data availability
- For clinical datasets or third party data, please ensure that the statement adheres to our policy

Full details of trait associations with variants and genes are available in Supplementary Data 1 and Supplementary Data 2, respectively. UKB phenotype data, genotyping array data, whole exome sequencing data, and whole genome sequencing data can be accessed via the UKB RAP: https://ukbiobank.dnanexus.com/

landing. All data used in this research are publicly available to registered researchers through the UKB data-access protocol and who are listed as collaborators on UKB-approved access applications. The HapMap3 reference panel was downloaded from ftp://ftp.ncbi.nlm.nih.gov/hapmap/. VCFs for TOPMED Freeze 8 were obtained from dbGaP as described in https://med.nhlbi.nih.gov/topmed-whole-genome-sequencing-methods-freeze-8. The human reference genome GRCh38 was obtained from http://ftp.1000genomes.ebi.ac.uk/vol1/ftp/technical/reference/GRCh38_reference_genome/.

Research involving human participants, their data, or biological material

Policy information about studies with human participants or human data. See also policy information about sex, gender (identity/presentation), and sexual orientation and race, ethnicity and racism.

Reporting on sex and gender

Sex of study participants was collected as part of the UK Biobank study as described in Bycroft et al, Nature 2018 (https://www.nature.com/articles/s41586-018-0579-z).

54.2% of the participants are women.

Sex was used as a covariate in the regression models for genetic association testing.

Reporting on race, ethnicity, or other socially relevant groupings

Genetic ancestry and continental ancestry assignments for each participant were inferred from the genotyping array and exome sequencing data as described in Backman et al, Nature 2021 (https://www.nature.com/articles/s41586-021-04103-z). To control for confounding due to genetic ancestry differences among the UK Biobank participants, 10 ancestry-informative principal components (PCs) derived from the whole exome sequencing variants were obtained and included as covariates in the regression models for genetic association testing.

Population characteristics

The details of the population characteristics of the UK Biobank are described in the paper by Bycroft et al, Nature 2018 (https:// www.nature.com/articles/s41586-018-0579-z). Briefly, 94.7% of participants are of European ancestry, 54.2% are female, the average age at assessment is 58. Each participant reports 8 inpatient ICD10 3D codes, on average.

Recruitment

Please see Bycroft et al, Nature 2018.

Ethics oversight

Ethical approval for the UK Biobank was previously obtained from the North West Centre for Research Ethics Committee (11/NW/0382). Informed consent was provided by all study participants. The work described herein was conducted under the approved UK Biobank application number 26041.

Note that full information on the approval of the study protocol must also be provided in the manuscript.

Field-specific reporting

Please select the one below that is the best fit for your research. If you are not sure, read the appropriate sections before making your selection.

☒ Life sciences ☐ Behavioural & social sciences ☐ Ecological, evolutionary & environmental sciences

For a reference copy of the document with all sections, see nature.com/documents/nr-reporting-summary-flat.pdf

Life sciences study design

All studies must disclose on these points even when the disclosure is negative.

Sample size

Sample sizes were not predetermined. To permit comparison of genetic association yield across platforms, the intersection of samples with array genotyping, exome sequencing, and genome sequencing data available was used, which yielded a sample of n=149,195 individuals after quality control (QC) filtering. See Methods section "UKB data preparation" for details on how the QC was performed. To evaluate the influence of sample size on genetic association discovery yield we also compared the primary analytical sample to a sample of n=468,169 individuals with both array genotyping and exome sequencing data available. A randomly selected sample of n=47,545 individuals with genome sequencing data was also used to assess the impact of sample size, where the size of this down sample was chosen to retain the same multiplier (3.138) between the aforementioned two larger sample sets. Lastly, we generated a replication sample by selecting all n=318,974 individuals in the UK Biobank with both array genotyping and exome sequencing data available and who were not included in the primary analytical set of n=149,195 individuals.

Data exclusions

Variant level QC was performed as described in methods section "Analysis-ready dataset preparation" Genotyping array and sequencing data was prepared for analysis using consistent filtering. Across all platforms,, we excluded variants with Hardy-Weinberg equilibrium (HWE) test P > 1 x 10-15 and >10% missingness. We further excluded all variants that failed QC in TOPMed Freeze 8 or gnomAD, as well as additional QC filters as described in more detail in the manuscript.

A set of 100 traits were selected from 492 traits that were previously described in Backman et al, Nature 2021 (https://www.nature.com/articles/s41586-021-04103-z). A total of 80 quantitative and 20 binary traits were selected by pruning for phenotype redundancy, sufficient case counts, and prioritizing trait heritability.

Replication

A sample of n=318,974 individuals from the UK Biobank with both array genotyping and exome sequencing data available and that were not included in the primary analytical set of n=149,195 individuals was used for replication of genome-wide significant associations identified in

	the primary analytical dataset. We replicated (a) 17 of 26 single variant and 14 of 18 gene-based signals only from WES+IMP, (b) 13 of 64 single variant and 5 of 10 gene-based signals only in WGS, and (c) 3,386 of 3,570 shared single variant signals and 514 of 556 shared gene-based signals.
Randomization	Individuals in the study were not being assigned to any experimental protocol or treatment and so randomization was not needed.
Blinding	Individuals in the study were not being assigned to any experimental protocol or treatment and so blinding was not needed.

Reporting for specific materials, systems and methods

We require information from authors about some types of materials, experimental systems and methods used in many studies. Here, indicate whether each material, system or method listed is relevant to your study. If you are not sure if a list item applies to your research, read the appropriate section before selecting a response.

Materials & experimental systems

n/a	Involved in the study
☒	☐ Antibodies
☒	☐ Eukaryotic cell lines
☒	☐ Palaeontology and archaeology
☒	☐ Animals and other organisms
☒	☐ Clinical data
☒	☐ Dual use research of concern
☒	☐ Plants

Methods

n/a	Involved in the study
☒	☐ ChIP-seq
☒	☐ Flow cytometry
☒	☐ MRI-based neuroimaging
